# Glioblastoma Masquerading as Metastasis in a Routine Follow-Up of a 79-Year-Old Woman

**DOI:** 10.7759/cureus.81231

**Published:** 2025-03-26

**Authors:** Rana Moshref, Abdurrahim Elashaal

**Affiliations:** 1 Neurosurgery, London Health Sciences Centre, London, CAN; 2 Neurosurgery, Windsor Regional Hospital - Ouellette Campus, Windsor, CAN

**Keywords:** butterfly glioma, glioblastoma, multicentric, multidisciplinary management, neurosurgery

## Abstract

Glioblastoma is the most aggressive and common primary brain tumor. Diagnosis is based on imaging and is confirmed through a brain biopsy. Multimodal treatment, including gross total resection, radiotherapy, and chemotherapy, is typically required. We report a case of a 79-year-old woman, a former smoker, who presented with a headache and generalized weakness and was found to have multiple brain lesions. The patient was diagnosed with glioblastoma and underwent partial resection. Multiple glioblastomas are a rare presentation and can be multicentric or multifocal. Patients with this presentation typically exhibit symptoms of high intracranial pressure. Glioblastomas have a poor prognosis despite multidisciplinary management. Glioblastoma should be considered a differential diagnosis for patients with multiple brain lesions.

## Introduction

Glioblastoma is the most aggressive and common primary brain tumor, constituting 13.9% of all tumors and 51.5% of all malignant tumors [1]. Patients typically present with nonspecific findings or symptoms of increased intracranial pressure such as vomiting, seizures, headaches, and visual changes. Imaging modalities include computed tomography (CT) and magnetic resonance imaging (MRI) of the head [2]. Glioblastomas are most commonly found in the frontal, temporal, parietal, and occipital lobes [3,4]. MRI of the head often shows “butterfly glioma tumors” involving the corpus callosum, temporal, and occipital lobes, characterized by hyperintense T2 brain lesions with surrounding edema [5,6]. Glioblastoma is an adult-type IDH wild-type diffuse astrocytic glioma and is diagnosed on molecular criteria as IDH wild-type based on the World Health Organization (WHO) 2021 report [7]. Multiple glioblastomas are a rare presentation, representing 12% of glioblastoma cases, and can be multicentric or multifocal [8]. We present a case of glioblastoma masquerading as metastasis in a 79-year-old female.

## Case presentation

**Figure 1:**
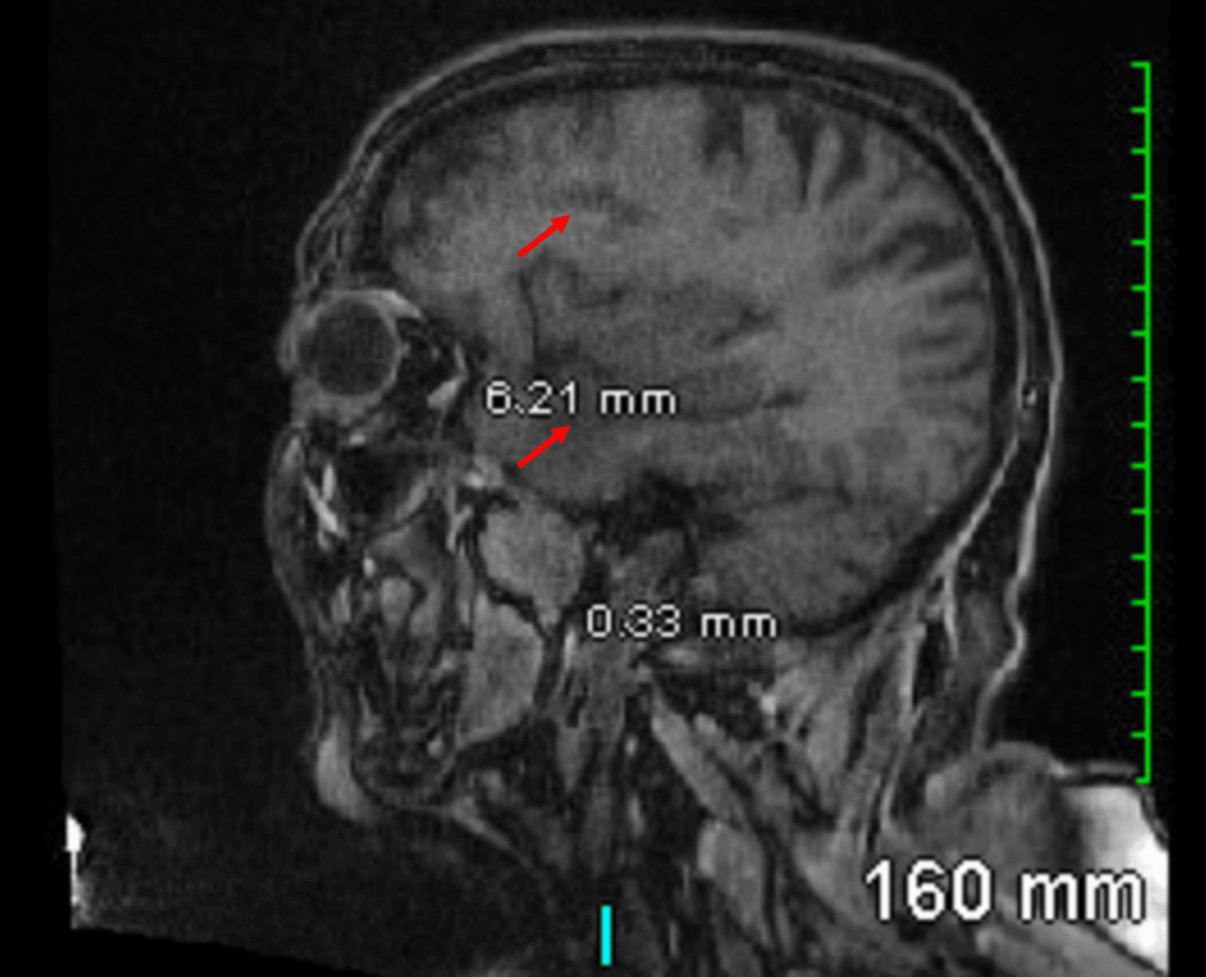
Preoperative MR head sagittal cut showing several new ring-enhancing lesions within the left temporal (5.6 x 3.5 cm), parietal (1.5 x 1.4 cm), and frontal (2.9 x 2 cm) lobes and the right parietal lobe (1.6 x 1.3 cm)

**Figure 2:**
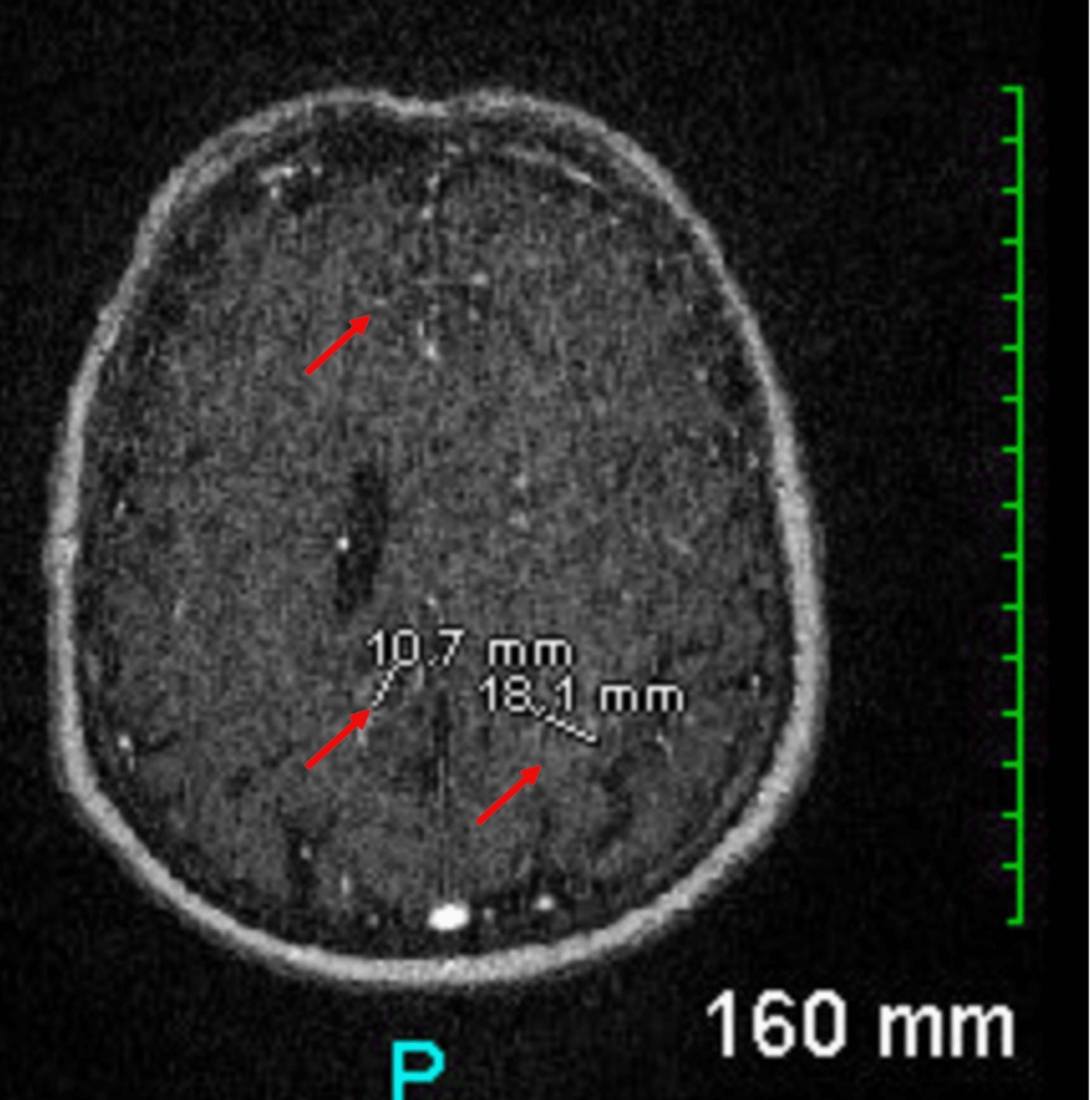
Preoperative MR head axial cut showing several new ring-enhancing lesions within the left temporal (5.6 x 3.5 cm), parietal (1.5 x 1.4 cm), and frontal (2.9 x 2 cm) lobes and the right parietal (1.6 x 1.3 cm) lobe

We report the case of a 79-year-old woman, a former smoker, who presented with a headache and generalized weakness for 2 months. She didn't seek medical attention and was not investigated. Four brain lesions were discovered during her routine yearly cerebral aneurysm follow-up MRI (Figures 1, 2).

The patient had no history of seizures. Her medical history included coiling, which means inserting coils into the aneursym through the arteries running from the groin to the head, a right carotid terminus intracranial aneurysm measuring 7 mm, a 2-3 mm left carotid terminus managed conservatively, migraines, hypertension, type 2 diabetes mellitus, depression/anxiety, dyslipidemia, obesity, appendectomy, hysterectomy, cesarean section, previous ductal carcinoma in situ, and fibromyalgia. She had no known medication allergies and denied any history of alcohol or recreational drug use.

The patient was alert and was having generalized weakness as part of managing the incidental brain lesions and her history of ductal carcinoma in situ. It was initially thought she had metastasis, for which a metastatic workup was ordered. There was no history of infectious causes and no recent travel history, so an infectious workup was not sent.

Given that metastasis was high on the differential, the patient was initiated on dexamethasone 2 mg twice daily, pantoprazole, and levetiracetam 500 mg twice daily. Her Karnofsky Performance Scale (KPS) was 70.

Investigations

Laboratory work was performed with unremarkable results. Imaging studies, including a CT chest, abdomen, and pelvis, MRI of the liver, and bone scan, were conducted. The CT chest and the bone scan were negative for metastasis. The CT abdomen and pelvis showed a hypoenhancing mass in the left lobe of the liver, measuring 2.8 x 1.5 x 2.5 cm, and several borderline portacaval lymph nodes, measuring 8-10 mm in the short axis. An MRI of the liver showed a 3.6 x 2.7 cm lesion within segment 4 with increased T2 signal and peripheral enhancement with no washout and multiple simple renal cysts noted measuring up to 1.2 cm.

Operative report

The patient underwent a left frontotemporal craniotomy for brain tumor resection of one of the six lesions (posterior frontal area). The patient underwent general anesthesia, and lines were connected. The patient was placed in a supine position, and a Mayfield clamp was applied for neuronavigation. A curved skin incision was made deep in the periosteum. Two burr holes were placed near the sinus on the left side, and a craniotome was used to connect them. The bone flap was elevated, the dura was opened, and slight maceration occurred. A corticectomy was performed, and samples were sent for frozen and permanent pathology. Frozen pathology was suggestive of a high-grade glioma. The left frontal lesion was resected, hemostasis was achieved, the dura was sutured with duraplasty, and the bone flap was secured with three cranial fixes. The skin was closed with Vicryl staples.

Pathology

The final pathological diagnosis was glioblastoma IDH1 R123H wild-type, WHO grade 4. Microscopically, the tumor exhibited hypercellularity, a fibrillary structure, pleomorphism, infiltration, and a patternless arrangement, with abundant mitotic activity, endothelial hyperplasia, and necrosis (pseudopalisading). Immunohistochemistry results showed positive glial fibrillary acidic protein (GFAP) (present), variable oligodendrocyte transcription factor 2 (OLIG2), alpha thalassemia/mental retardation syndrome X-linked gene (ATRX) (interpreted as retained-wildtype), IDH1-R123H mutation (absent), p53 (increased expression >10% of lesional cells suggesting mutation), Ki67 (highly variable but focally up to 40%), MLH1/MSH2, MSH6, and PMS2 (all somehow variable, interpreted as retained).

Postoperative hospital course

**Figure 3:**
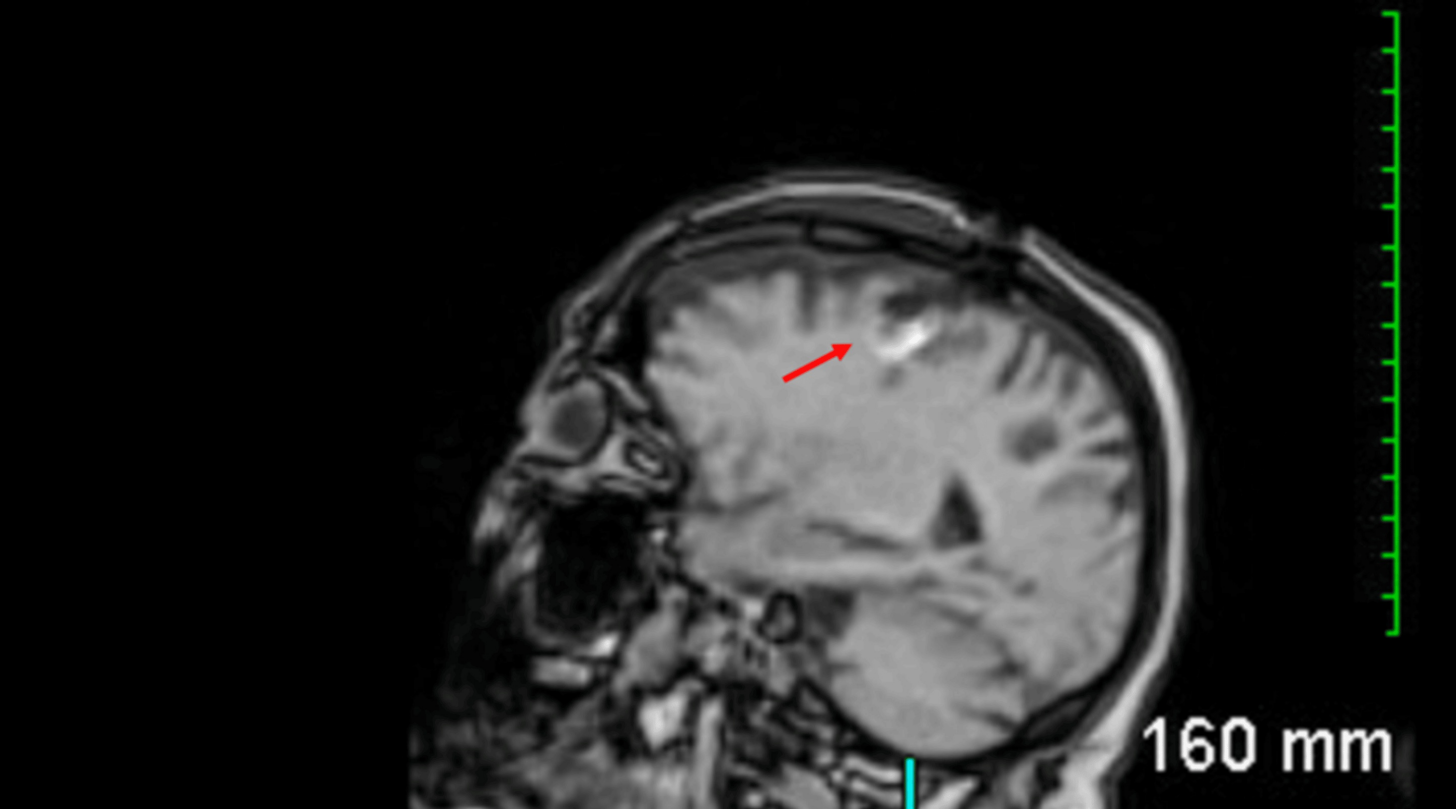
Postoperative MR head sagittal cut shows postsurgical changes

Postoperatively, the patient exhibited no gaze palsy, neglect, or facial asymmetry. She was able to move all four extremities and respond intermittently to commands. Her generalized weakness didn't improve with steroids or surgery. Her neurological exam fluctuated throughout her hospital stay. Her KPS postoperatively was 30. A CT head scan revealed postsurgical changes with pneumocephalus, and an MRI confirmed these postsurgical changes (Figure 3).

During her hospital stay, she experienced altered levels of consciousness and seizures. Her levetiracetam dose was increased to 1 g twice daily, and lacosamide 50 mg twice daily was added. Both medical and radiation oncology teams assessed her condition and provided a prognosis of three to six months due to her advanced age and multifocal disease. The patient was scheduled for palliative external beam radiotherapy to the whole brain of 20 Gray (Gy)/5 fractions over 5 days. She was diagnosed with hyponatremia and treated with 3% normal saline. She was transferred to hospice care after completing the goals of the care meetings. Three weeks after admission, she was following simple instructions, speaking one to two words, and exhibited right-sided weakness because of the progression of the disease.

## Discussion

Multiple glioblastomas are classified based on disease infiltration into the commissural fibers, cerebrospinal fluid, and direct extension [9]. Multicentric glioblastomas are defined as two or more masses at least 2 cm apart. Patients with this presentation typically exhibit symptoms of high intracranial pressure [10]. Poor predictors in multicentric glioblastoma included age more than 60 years old, subtotal resection, multiple lesions, and not receiving adjuvant radiation therapy [11]. No significant differences have been found in methylation or amplification between solitary and multiple glioblastomas [8,12].

Rapid early progression (REP) occurred in nearly half of the diagnosed cases of glioblastoma in general, and no study studied REP in multicentric glioblastoma. REP is defined as an increase in enhancement, vascularity, and new enhancing lesions with or without restricted diffusion. It is a negative predictor that does not correlate with methylation status [13]. It usually presents in the frontal lobe, followed by other lobes, and is typically infiltrative [3,14,15]. There is a similar case to ours; that case report was about a 60-year-old man with multiple brain lesions who was admitted with confusion and started on Mannitol and Tegretol. He was initially treated for positive Schistosoma mansoni test results but was later diagnosed with glioblastoma. His health deteriorated eight months after diagnosis [14]. Other cases of multiple glioblastomas present with sudden loss of consciousness, headache, seizures, and weakness [15]. The differential diagnoses of multiple brain lesions include abscess, infarct, metastasis, contusion, glioblastoma, radiation necrosis, demyelinating disease, and hematoma [16].

Multimodal treatment, including radical resection, radiotherapy, and chemotherapy, is required to improve survival in multicentric glioblastoma, with a median survival of eight months [8,11,17]. Despite multidisciplinary management, multicentric glioblastomas have a poor prognosis. Poor prognostic factors include high contrast enhancement, hemorrhage, edema, and rapid early progression [6,8]. Temozolomide therapy works on DNA repair protein O6-methylguanine-DNA methyl-transferase (MGMT) and is correlated with the methylation status of the tumor [17,18].

## Conclusions

Glioblastomas have a generally poor prognosis despite multidisciplinary management, and multicentric glioblastoma has an even worse prognosis. When multiple brain lesions are observed, glioblastoma should be considered in the differential diagnosis. To increase survivability, multimodal treatment, including radical resection with adjuvant chemoradiation therapy, is recommended. Multidisciplinary meetings and discussions on the goals of care should be conducted.
